# Agar Contact Method as a Valuable Tool to Identify Slaughter Hygiene Deficiencies along the Slaughter Process by Longitudinally Sampling Pig Skin Surfaces

**DOI:** 10.3390/microorganisms11102512

**Published:** 2023-10-08

**Authors:** Roland Fürstenberg, Nina Langkabel, Julia Grosse-Kleimann, Lothar Kreienbrock, Diana Meemken

**Affiliations:** 1 Working Group Meat Hygiene, Institute of Food Safety and Food Hygiene, School of Veterinary Medicine, Freie Universität Berlin, 14163 Berlin, Germany; roland.fuerstenberg@fu-berlin.de (R.F.);; 2 Department for Biometry, Epidemiology and Information Processing, WHO Collaborating Centre for Research and Training for Health at the Human-Animal-Environment Interface, University of Veterinary Medicine Hannover, Foundation, 30559 Hannover, Germany

**Keywords:** swine, slaughterhouse, microbial load, *Salmonella*, repeated sampling, agar contact plates

## Abstract

Examinations of total viable counts (TVCs) and *Salmonella* spp. on the skin of individual pigs during the slaughter process are useful to identify abattoir-specific risk factors for (cross-)contamination. At seven process stages (lairage to before chilling), pigs were bacteriologically investigated by repeatedly sampling the same animals using the agar contact method. The mean TVC of all pigs increased significantly at the first three tested process stages (mean count, after delivery: 5.70 log cfu/cm^2^, after showering: 6.27 log cfu/cm^2^, after stunning: 6.48 log cfu/cm^2^). Significant mean TVC reductions occurred after scalding/dehairing (mean count: 3.71 log cfu/cm^2^), after singeing/flaming (2.70 log cfu/cm^2^), and after evisceration (2.44 log cfu/cm^2^) compared with the respective preceding process stages. At the end of the slaughter line and before chilling, the mean TVC was 2.33 log cfu/cm^2^, showing that the slaughter process reduced contamination significantly. The slaughter process effectively reduced even very high levels of incoming TVCs, since at the individual animal level, at the end of the slaughter process, there was no difference in the TVCs of animals with initially high and initially low TVCs. Additionally, 12 *Salmonella* spp. isolates were recovered from 12 different pigs, but only until the stage after scalding/dehairing. Overall, the agar contact method used is valuable for detecting hygiene deficiencies at slaughter, and is animal-equitable, practical, and suitable for use on live animals.

## 1. Introduction

Initial bacterial contamination, cross-contamination, and re-contamination can be identified on carcasses at different stages during the slaughter process, meaning that the resultant meat can pose a risk factor for consumer health [1]. Therefore, in the European Union, food business operators must ensure that their products comply with microbiological criteria for food safety and process hygiene as laid down in Regulation (EC) No 2073/2005 [2,3]. In order to identify unacceptable or critical levels of bacterial loads on carcasses, the microbiological risk management process is based on monitoring procedures [4]. For this purpose, on pig carcasses, the total viable count (TVC) of aerobic mesophilic bacteria, *Enterobacteriaceae* counts, and tests for the presence/absence of *Salmonella* spp. (*S.*) are required as hygiene indicators [3,5,6]. In particular, the TVC is most frequently used to evaluate the hygiene of the entire meat production process [7,8] and can provide a record of the overall hygienic performance of the slaughter process over time [9]. Additionally, testing for *Salmonella* spp. is of utmost importance because of its zoonotic potential, and since pigs are regarded as one of the most common foodborne sources of human *Salmonella* spp. infections [10,11,12]. Over 70% of the contamination with *Salmonella* spp. on a carcass originates from the slaughter pig itself, while 30% of the contamination is related to cross-contamination by carrier pigs in the slaughter line [13]. In addition, risks for bacterial contamination of carcasses can include the repeated introduction of bacteria into the abattoir via the animals, the equipment being contaminated during improper slaughter practices, and cross-contamination by resident bacteria during the slaughter process [14]. Furthermore, multidrug resistance has increased all over the world and is considered a public health threat [15]. Several recent investigations reported the emergence of virulent multidrug-resistant bacterial pathogens from different origins that increase the necessity of the proper use of antibiotics as well as the application of rapid accurate diagnostic tools for the screening of the emerging virulent multidrug-resistant strains [16,17,18]. Therefore, sampling at various process stages could be useful to identify hygienic deficiencies.

In accordance with the EN ISO 17604:2015-12 standard protocol [19], sampling methods for carcasses are divided into destructive and non-destructive methods. Various studies determined tissue excision, a destructive method, to be the method with the highest precision compared to non-destructive methods [20,21]. However, in the case of repeated sampling from the same pig carcass in order to identify deficiencies in process hygiene, tissue excision leads to substantial loss of the carcass surface [22]. Because of the proportional relationship between the lower microbiological recovery by using non-destructive methods compared with the destructive method, the results obtained using non-destructive methods can be also valuable in determining the slaughter hygiene [23]. For this reason, commercial abattoirs in Europe often use the non-destructive method of wet–dry double swabbing for monitoring [24]. Additionally, contact methods, as non-destructive methods, are used for semi-quantitative microbiological examination of the environment, fitments, equipment, and utensils [25]. To enable quantitative counting of bacteria, a modified microbiological procedure was developed and called the agar contact method [26,27]. Moreover, the suitability of contact methods for the microbiological sampling of carcass surfaces has already been proven [28]. Previous comparative investigations between the contact methods and the wet–dry double swabbing method on pig carcass surfaces showed that the wet–dry double swabbing method recovered TVCs that were only up to 0.5 log levels higher compared to the method using agar contact plates [29,30]. Differences of up to 0.5 log levels are within the normal laboratory range and can be regarded as similar [31], so the practical importance of this minor mean difference in TVC is questionable [32]. Our previous investigation on pig carcasses also showed that the agar contact method yielded comparable results to the wet–dry double swabbing method [33].

Major advantages of the agar contact method are that it is a fast, practical, and animal-equitable sampling technique that can be used during routine slaughter, including in the lairage, without interrupting the work process. Additionally, the agar contact method is suitable for the repeated sampling of the same animal and its resulting carcass at different process stages. Milios et al. (2014) [23] concluded that microbiological results measured at various stages of the process should be used to provide knowledge on the cause of possible problems. There are relatively few investigations spanning the pig slaughter line at all process stages, starting in the lairage and ranging to the end of dressing (before chilling) [34]. These abattoir-specific data, with additional assessment of the associated health risk potential, provide the basis for implementing an effective monitoring system [35].

The aim of this study was to examine the skin of live slaughter pigs and their respective carcasses longitudinally over the course of the slaughter process from the lairage to the end of the slaughter line to quantify the bacterial loads and to detect *Salmonella*-positive pigs. For this special case investigation, we used the agar contact method to realize the repeated sampling of individual pigs at all seven process stages. The trend of the TVC loads of all pigs over the course of the slaughter line was examined to identify process stages with possible risk factors for carcass contamination, and in addition, the effect of the process was investigated at the individual pig level and considering the pigs with the highest and the lowest incoming TVC loads.

## 2. Materials and Methods

### 2.1. Slaughter Line

The study was conducted at an industrial pig abattoir located in Northwestern Germany with a slaughter capacity of 2900 pigs per day. After arriving at the abattoir, slaughter pigs were showered for 5 min with water to cool and calm them after transport. After a resting time in the lairage of approximately one hour, they were driven to the carbon dioxide stunning system (Butina A/S, Holbæk, Denmark), where they were stunned and immediately bled in a hanging position with blood-draining knives in a rotating carousel (Anitec AB, Malmö, Sweden). After pre-washing, a combined process stage followed where the carcasses were scalded in a steam tunnel at 63.5 °C (BANSS Schlacht- und Fördertechnik GmbH, Biedenkopf, Germany) and immediately afterwards dehaired using rotating scrapers/rubber whips (BANSS Schlacht- und Fördertechnik GmbH, Biedenkopf, Germany). Singeing/flaming was carried out in two successive ovens, with wet polishing between both ovens (BANSS Schlacht- und Fördertechnik GmbH, Biedenkopf, Germany). After manual rectum cutting with a cutter (Schmid & Wezel GmbH, Maulbronn, Germany) and evisceration, the carcasses were split using a manually guided water-cooled splitting saw (Schmid & Wezel GmbH, Maulbronn, Germany), followed by trimming steps and official meat inspection before entering the chilling area.

### 2.2. Sampling Procedure of Pig Skin and Carcass Surface

In order to examine the effect of the slaughter process on the microbial level and to identify specific process hygienic deficiencies, seven different process stages were identified at which the pigs (identified by individual marking) were sampled: 1. lairage before showering; 2. lairage after showering; 3. after stunning; 4. after scalding/dehairing; 5. after singeing/flaming; 6. after evisceration; and 7. before chilling.

Altogether, 87 conventionally raised fattening pigs were randomly chosen from ten different selected batches (i.e., nine individual pigs per batch for nine batches, and six individual pigs for one batch; the pigs were numbered from Pig ID 1 to 90, but three pigs of an unknown batch with the Pig ID 64, 65, and 66 were finally removed, resulting in 609 samples) and were sampled at the seven abovementioned process stages. During five visits between May and August 2022 (Wednesday: batches No 1, 2; Monday: batches No 3, 4; Tuesday: batches No 5, 6; Thursday: batches No 7, 8; Friday: batches No 9, 10), one visit per day was conducted, and pigs from two batches per day were sampled longitudinally along the slaughter line. There was a time interval of approximately one hour between the sampling of the two batches on every sampling day. For individual identification, in the lairage, the pigs were marked on their backs using a livestock pen (Schippers GmbH, Kerken, Germany), and after the stunning and after bleeding, the carcasses in the study were marked by a tattoo identification number using a tattoo marker and black ink (R & M Horn Farmservice Co. KG, Dortmund, Germany).

**Figure 1:**
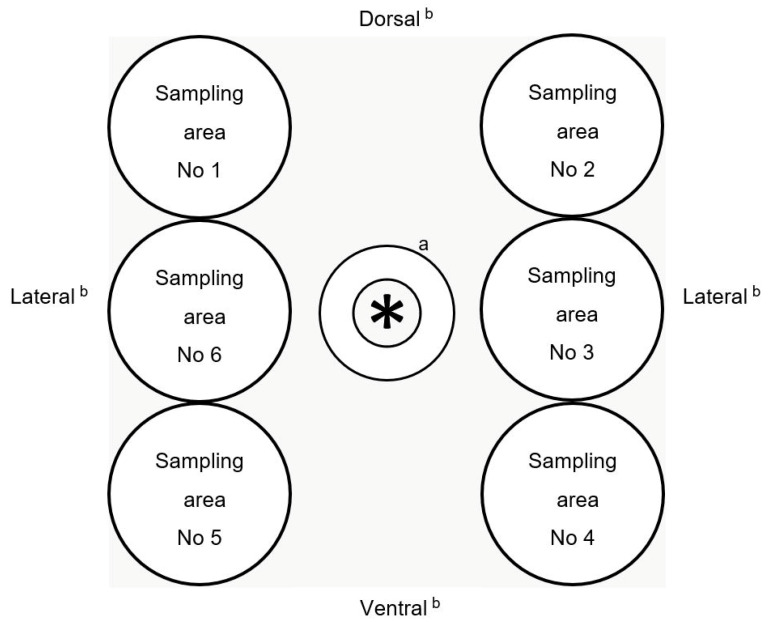
Schematic diagram of the sampling areas in the perianal region with the view of the sample taker from behind the standing live slaughter pig and the pig carcass; ^a^ = anatomical localization/position of the anus; ^b^ = anatomical direction.

Sampling was performed by using commercial agar contact plates containing plate count agar with a raised contact surface of 23 cm^2^ (VWR International GmbH, Darmstadt, Germany). For sampling, six areas around the anus were defined (three separate areas each of approximately 23 cm^2^ directly left and three separate areas directly right from the anus). From one process stage to the other, a different area was sampled in a rotating manner for every individual pig, starting from sampling area 1 for the first pig and sampling area 2 for the second pig, etc. (Figure 1). Differing from this pattern of sampling, for process stage 4 (after scalding/dehairing), we used the same sampling area from where we had taken the sample for process stage 1 on each individual pig carcass, meaning sampling area 1 was sampled twice for the first pig, sampling area 2 was sampled twice for the second pig and so on. For repeated sampling of the same pig at different process stages, to take one sample, the cover of an agar contact plate was removed directly in front of the pig skin, and each agar contact plate was pressed firmly for 5 s against the perianal area without lateral movement and using constant, even pressure. The procedure was performed for all pigs in the lairage pen and for each pig’s respective carcass. After sampling, the plates were transported to the institute’s laboratory in Berlin at 4 °C and processed within 24 h. During sampling using the agar contact plates in the perianal area, i.e., pressing the soft nutrient medium onto the skin, the live and free-running slaughter pigs in the lairage pens did not show any reaction, and furthermore, no restraint of the animals was required.

### 2.3. Microbiological Analysis

For microbiological analysis of TVC via the drop plating method and for qualitative analysis of *Salmonella* spp., in contrast to the common contact method, each agar slice of the contact plates was dislodged from the Petri dish using sterile forceps and transferred into a blender bag with a filter (VWR International GmbH, Darmstadt, Germany). Afterwards, 100 mL of buffered peptone water broth (Merck KGaA, Darmstadt, Germany) was added. According to German standard DIN 10161:2016-12 [36], the content of the blender bag was homogenized with a stomacher (bioMérieux, Marcy-l´Étoile, France) for two minutes with a speed of 560 strokes per minute. The resulting basic homogenate was used as a basis for TVC and *Salmonella* spp. detection.

For quantitative analysis of TVC, decimal dilution series were prepared from each basic homogenate with sodium chloride peptone agar (Merck KGgA, Darmstadt, Germany), using tenfold decimal dilutions up to 10^−6^ dilution. From basic homogenate and each dilution series, duplicate 0.05 mL amounts were dropped onto plate count agar (Th. Geyer GmbH & Co. KG, Renningen, Germany) and streaked out using a loop (Sarstedt AG & Co. KG, Nümbrecht, Germany). Plates were incubated under aerobic conditions at 30 °C for 72 ± 2 h. Following this incubation, colonies were counted on each dilution step. Colonies were also counted in the case where only one colony had developed in the minimum dilution or in basic homogenate. Afterwards, the weighted arithmetic mean for each sample was calculated.

### 2.4. Isolation and Identification of Salmonella spp.

Qualitative analysis of *Salmonella* spp. was performed according to EN ISO 6579-1:2020-08 standard protocol [37]. The basic homogenate, which contained the agar slice and 100 mL of buffered peptone water broth (Merck KGaA, Darmstadt, Germany), homogenized with a stomacher, provided the basis for pre-enrichment. Deviating from the ISO standard protocol after incubation of the basic homogenate at 37 °C for 24 h for pre-enrichment, enrichment broths, Rappaport Vassiliadis Bouillon (RV) (Merck KGaA, Darmstadt, Germany) and Muller–Kauffmann Tetrathionate-Novobiocin Broth (MKTTn) (Merck KGaA, Darmstadt, Germany) were incubated for 24 h at 37 °C for MKTTn and at 42 °C for RV, before streaking out on the two selective agars, Brilliant-Green Phenol-Red Lactose Sucrose Agar (Merck KGaA, Darmstadt, Germany) and Miller and Mallinson ChromoSelect Agar (Sigma-Aldrich, St. Louis, MO, USA). Plates were incubated for 24 h at 37 °C. Biochemical testing for dulcitol, o-nitrophenyl-β-D-galactopyranoside (ONPG), malonate, and salicin was carried out for further investigation of the isolates. Typical and suspect colonies were tested using agglutination tests (sifin diagnostics gmbh, Berlin, Germany), and *Salmonella* spp. confirmation was carried out using polymerase chain reaction tests, performed at the German Federal Institute for Risk Assessment (BfR) in Berlin, Germany.

#### Minimal Inhibitory Concentration (MIC)

The recovered *Salmonella* spp. isolates were tested for their susceptibility to a fixed panel of antimicrobials using the broth microdilution method, following the Clinical and Laboratory Standards Institute (CLSI) guidelines [38]. The MIC testing was performed at the German Federal Institute for Risk Assessment (BfR) in Berlin, Germany, and in this study, the breakpoints for susceptible, intermediate, and resistant to the antimicrobial agents for *Enterobacteriaceae* were used, which were determined according to the performance standards for antimicrobial susceptibility testing by CLSI [39].

### 2.5. Statistical Analysis

For statistical analyses, TVC outcomes were transformed via logarithm to the base of ten, since the original values in colony forming units per square centimeter (cfu/cm^2^) showed a skewed distribution. The minimum limit of detection (one colony counted only) was 1.94 log cfu/cm^2^, and in the case of no visible growth, for statistical test purposes, bacteria counts were considered as half of the minimum limit of detection [40,41], which was 1.64 log cfu/cm^2^ for TVC.

First, descriptive analyses were conducted on both the pig batch and individual pig levels to show the distribution of log TVC at the different stages. In order to calculate the effect of the process stage, batch, and perianal sampling area on log TVC, separate analyses of variance (ANOVA) were conducted. Afterwards, the microbiological status between the different stages was examined in a mixed model with batch as a random effect and individual pig variations as repeated measurements.

A line graph of the five animals with the highest and lowest incoming bacterial loads (lairage before showering) on the skin, respectively, was created to visualize the reduction effect of the slaughter process. Only individual pigs with complete data from all stages were involved.

The influence of the purely mechanical part of the slaughter process on TVC was investigated via *t*-test by comparing the reduction that occurred between the lairage before showering up to the singeing stage between the 10% of slaughter pigs with the initially (lairage before showering) highest TVC loads and all other animals sampled. For these analyses, only stage 1 (lairage before showering), considered as incoming bacterial load, and stage 5 (after singeing/flaming), considered as last mechanical part of the slaughter line, were taken into consideration.

Results of *Salmonella* spp. were recorded as present or absent for each live slaughter pig and its respective carcass at all process stages.

All statistical analyses were performed with SAS (SAS^®^, version 9.4, SAS Institute Inc., Cary, NC, USA).

## 3. Results

The focus of our work was to examine the status quo of the contamination level on the slaughter pig surface and the respective carcass surface by means of longitudinal sampling of the same pigs at seven process stages along the entire slaughter line. We wanted to show, via this study, the trend curve of surface contamination on slaughter pigs, starting after delivery of the animals in the lairage pens at the abattoir up to the process stage before chilling, to investigate whether the slaughter process reduces the pig surface contamination level in a meaningful way and to identify possible slaughter hygiene deviancies over the course of the slaughter line.

### 3.1. Descriptive Analysis of Total Viable Count at Batch Level

Six samples from four batches were unavailable, resulting in 603 samples being considered for analysis.

**Figure 2:**
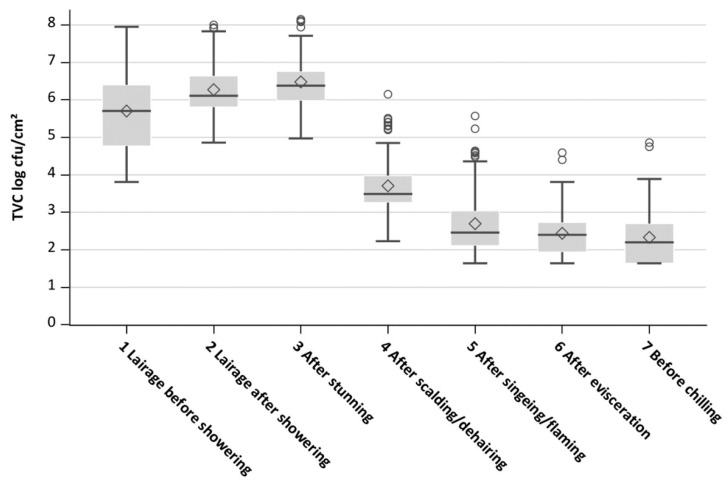
Boxplots of the mean logarithmic total viable counts (TVC log cfu/cm^2^) of all pig batches at seven process stages: 1 lairage before showering; 2 lairage after showering; 3 after stunning; 4 after scalding/dehairing; 5 after singeing/flaming; 6 after evisceration; 7 before chilling.

The mean TVC level of all batches at the first process stage in the lairage was 5.70 log cfu/cm^2^ and then increased up to 6.48 log cfu/cm^2^ after stunning. After this stage, the TVC of the carcasses reduced to 2.70 log cfu/cm^2^ after singeing/flaming and reduced further to 2.33 log cfu/cm^2^ before chilling (Table 1). Standard deviations ranged between 0.64 (after evisceration) and 1.05 (lairage before showering) log cfu/cm^2^. The boxplots in Figure 2 visualize the decrease in variation after the first process stage and the noticeable reduction in TVC after scalding/dehairing.

**Figure 3:**
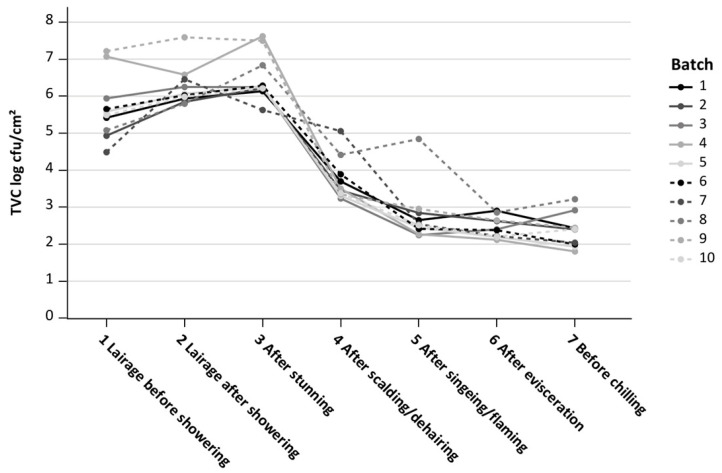
Trend curves of the mean logarithmic total viable count (TVC log cfu/cm^2^) for each of the ten pig batches at seven process stages: 1 lairage before showering; 2 lairage after showering; 3 after stunning; 4 after scalding/dehairing; 5 after singeing/flaming; 6 after evisceration; 7 before chilling; Batch = number of pig batch.

Figure 3 shows that the mean TVCs of each pig batch were close together in the range of approximately 1.50 log levels. In contrast to this, batches No 4 and No 9 both had a high incoming TVC of above 7 log cfu/cm^2^ (lairage before showering), but this decreased to the log level of the other batches at process stage 5 (after singeing/flaming). At this stage, the mean TVC of batch No 8 (containing only six individual pigs compared to the nine pigs of all other batches) increased to 5 log cfu/cm^2^, which was around 2 log levels above the other batches, and then decreased in the range of log levels of the other batches after evisceration.

### 3.2. ANOVA of Total Viable Count at Batch Level

Modelling TVC log cfu/cm^2^ only by process stage and together with batch as an interaction factor, respectively, both resulted in a statistically significant influence (*p* < 0.0001). However, for perianal areas, no differences were detected (*p* = 0.8139). The results of the mixed model with stage as a fixed effect, batch as a random effect, and individual pig variations are shown in Table 2. The TVC of pigs increased statistically significantly after process stage 1 (lairage before showering), first by 0.57 log cfu/cm^2^ (*p* < 0.0001) after showering and then again slightly after stunning by 0.21 log cfu/cm^2^ (*p* = 0.0466). After process stage 4 (scalding/dehairing), the TVC of the carcasses decreased by 2.78 log cfu/cm^2^ (*p* < 0.0001) and again by 1.00 log cfu/cm^2^ (*p* < 0.0001) after process stage 5 (singeing/flaming). After evisceration and before chilling, only small reductions of 0.26 log cfu/cm^2^ (*p* = 0.0139) and 0.11 log cfu/cm^2^ (*p* = 0.2891) occurred, respectively. The latter reduction was not statistically significant.

### 3.3. Total Viable Count at Individual Pig Level

**Figure 4:**
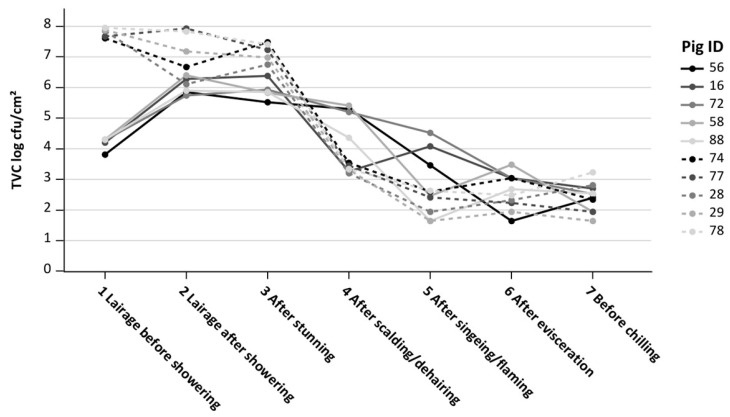
Trend curves along the slaughter line of the five pigs with the highest and five pigs with the lowest initial total viable counts (TVC log cfu/cm^2^) in the lairage before showering; Pig ID = number of the individual pig.

On an individual animal basis, the pigs with the five highest and five lowest incoming TVCs in the lairage were considered. The five pigs with the highest incoming TVC at lairage before showering, 7.50 to 8.00 log cfu/cm^2^, had similar outgoing loads before chilling, 1.70 to 3.20 log cfu/cm^2^, as did the five pigs with the lowest incoming TVCs. After scalding/dehairing, four of the five carcasses with low incoming TVC, 4.40 to 5.50 log cfu/cm^2^, had TVCs above those of the five initially highest TVC carcasses, which had TVCs of 3.10 to 3.50 log cfu/cm^2^ after scalding/dehairing (Figure 4).

### 3.4. Reduction Effect of Mechanical Stages

Variance homogeneity was first tested (Folded F = 0.0003), which is why the Satterthwaite method for unequal variances was used. The mean reduction in TVC of the 10% of pigs with the highest initial TVC (process stage 1: lairage before showering) to the last mechanical stage of the slaughter process (process stage 5: after singeing/flaming) was 5.11 log cfu/cm^2^, while the remaining animals showed a mean decrease of 2.75 log cfu/cm^2^. By means of a *t*-test, the mean difference of −2.36 log cfu/cm^2^ (difference between the mean reduction after singeing/flaming of the 10% of pigs with the highest incoming load and the other 90% of pigs) was shown to be statistically significant (*p* < 0.0001).

### 3.5. Salmonella spp. Occurrence

In total, 2% of all samples (12/603) were *Salmonella*-positive. These samples belonged to 12 different pigs from four batches. *Salmonella*-positive samples were only detected at the process stages between lairage and after scalding/dehairing. Table 3 gives an overview of the *Salmonella*-positive samples and detected serovars per individual positive pig, batch and process stage.

### 3.6. Phenotypic Characteristics of the Recovered Salmonella Spp. Isolates

The following serovars were detected in the 12 *Salmonella*-positive samples: monophasic *S.* Typhimurium 1,4,[5],12:i:- (n = 4), monophasic O-antigen-negative *S.* Typhimurium 1,4,12:i:- (n = 7), and monophasic *Salmonella* spp. 9,12:l,v:-. All four monophasic *S.* Typhimurium 1,4,[5],12:i:- were typically dulcitol-positive, and negative results for ONPG, malonate and salicin were observed in the biochemical testing.

The four monophasic *S.* Typhimurium 1,4,[5],12:i:- isolates were resistant to the antimicrobial agents ampicillin and sulfamethoxazole. All seven monophasic O-antigen-negative *S*. Typhimurium 1,4,12:i:- were resistant to ampicillin, sulfamethoxazole, and tetracycline. Monophasic *Salmonella* spp. 9,12:l,v:- (n = 1) was resistant to ciprofloxacin, nalidixic acid, and sulfamethoxazole. The results of MIC testing for antimicrobial susceptibility testing of the recovered *Salmonella* spp. isolates are shown in Table 4.

## 4. Discussion

Overall, the slaughter line investigated here reduced the variation in TVC values through the different process stages, but the analysis of the individual pigs also showed individual TVCs differing from the standard deviation of the average for all pigs. The trend of the variation of TVC values over the course of the slaughter line agrees with examinations by Schertenleib et al. (2011) [42].

After showering and resting of the slaughter pigs in the lairage, a statistically significant increase in microbiological load on the skin was found. Possible factors contributing to the increasing TVC could be that the shower water loosens dry dirt on the pig’s skin, and additionally, within a resting time up to one hour, pigs could shed feces and lay down in contaminated areas of the lairage pens. The fecal shedding of pigs can lead to pre-slaughter skin cross-contamination via contact between the slaughter pigs or between the contaminated environment and the pigs during the stay in the lairage [43]. Similar to our results, a study investigating *Enterobacteriaceae* showed a statistically significant increase in the *Enterobacteriaceae* count after 30 min of continuous water misting on slaughter pigs [44]. In our study, the highest TVC values were determined after stunning and bleeding, and this is line with previous studies covering the pig slaughter line [42,45]. Stunning leads to slackening of the sphincter muscles, which can result in leakage of feces from the rectum [46]. However, the increase of 0.21 log cfu/cm^2^ from lairage after showering to after stunning was much less than a 0.5 log level, which is within the normal variation range of classical microbiology laboratory analyses [31]. The practical importance of this minor mean difference in TVC between the two process stages is questionable [32]. After showering and after stunning, where statistically significant TVC increases were shown, the process stages should be assessed as stages with a potential risk of cross-contamination. Scalding/dehairing and singeing/flaming led to significant reductions in TVC, presumably moderated by the ideal duration and optimal temperature in the scalding condenser and in the singeing machine [47,48]. This was confirmed especially by the TVC reductions after singeing/flaming of batches No 4 and No 9, which both arrived in the abattoir lairage with very high TVCs. The combined scalding and dehairing reduced the mean TVCs compared to the TVCs after stunning/bleeding by approximately 2.7 log cfu/cm^2^, which is in accordance with the results from Schertenleib et al. (2011) [42]. Another European study using sponges for sampling showed a comparable trend of TVC values along the slaughter line as in our results [34]. The current study showed that TVC values significantly reduced after scalding and singeing and agrees with various other studies [34,35,49,50]. Furthermore, the TVC values underwent a minor reduction after evisceration and the stage before chilling, which is in line with an Irish study [34].

Possible factors causing the small increase in TVC variation at the end of the slaughter line could be the cross-contamination of carcasses by equipment and utensils. In pig meat processing, evisceration is recognized as a critical step that often results in carcass contamination [51,52]. Differences between abattoir staff in the hygienic performance of evisceration could contribute to differences in the cross-contamination of pig carcasses and in the probability for the transfer of pathogens to the meat [51,53]. Overall, and in agreement with other studies [34,42], we showed that the pig slaughter process seems to harmonize the variations of the TVC by effectively reducing the bacterial load to similarly low values.

The results of the mixed model with the process stage as a fixed effect, the batch as a random effect, and individual pig variation confirmed the findings of the effects of most process stages, with statistically significant differences in TVCs when comparing each stage to its following stage, but not between evisceration and before chilling.

Notably, (1) some TVCs were low, i.e., just inside the minimum detection limit of our method, (2) contact methods are suitable for microbiological sampling of carcass surfaces [28], but produce TVCs up to 0.5 log levels lower than wet-dry double swabbing [29,30], and (3) differences of up to 0.5 log levels are within the normal laboratory range [31]. The agar contact method is not listed in the EN ISO 17604:2015-12 standard protocol for carcass sampling for microbiological analysis [19]. Contact plates are commonly used for the microbiological examination of environmental surfaces [54], and the contact method is laid down in the EN ISO 18593:2018 [55] standard protocol. However, to realize our longitudinal examination without stopping the slaughter line or reducing the line speed, neither of which were acceptable, as we wanted to obtain a realistic picture of the actual slaughter process, and so we selected the agar contact method for the following important reasons. The selected procedure enabled the repeated sampling of individual slaughter pigs at seven process stages without interruption of the workflow. Additionally, this animal-equitable, fast, and practical sampling method can be used for sampling live pigs in the lairage without fixation of the pigs and also for carcass surfaces. Then, for this procedure using commercial agar contact plates, only a few further materials are required [56].

In order to avoid a reduction in skin contamination caused by repeated sampling with contact plates, six sampling areas around the anus were defined and a different area was systematically used for every pig at each process stage. In this study, no statistically significant differences for the changing perianal area between the individual stages were detected, and thus, the samples were considered comparable. In addition, this result and the possible distribution of contamination after showering in the lairage are the reasons why we chose the area from process stage 1 (lairage before showering) for resampling at process stage 4 (after scalding/dehairing) and considered the effect of the removal of the contamination from the same sampling area as very low.

However, batch No 8 showed higher TVCs after singeing/flaming, but similar TVCs after evisceration, compared to the other batches. Since the effectiveness of singeing/flaming on bacterial reduction differs depending on the system [47,48,57], it is possible that the carcass surfaces of batch No 8 were insufficiently processed by the ovens. The TVC reduction after evisceration could be explained by contamination on the perianal area being distributed to the front half of the carcass by the water used by the water-cooled splitting saw and, therefore, not being in the sampled area any longer. This saw is located after evisceration, but before trimming. In agreement with our results, reductions in *Enterobacteriaceae* after the evisceration of pig carcasses were shown previously [48]. Our results could also simply highlight the effect of individual batches in a slaughter process scenario during regular, routine work and with a representative sample of slaughter pigs. More in-depth studies are needed to resolve this point.

When considering individual pigs at slaughter, it was seen that regardless of the incoming microbiological load, the process harmonizes the outgoing TVC loads at the end of the slaughter line (before chilling) to a similar range. This was shown by our consideration of the five pigs with the highest compared to the five pigs with the lowest incoming TVCs.

Since we were able to detect several *Salmonella* spp. serovars on individual pig carcasses on the same sampling days, it could be that the pigs became contaminated with this pathogen via feces containing *Salmonella* spp. from contaminated lairage pens [58]. On the Tuesday, four monophasic *S.* Typhimurium 1,4,[5],12:i:- (all from batch No 6) were found. Additionally, on the Wednesday, seven O-antigen-negative monophasic *S.* Typhimurium 1,4,12:i:-, five from batch No 2 and two from batch No 1, were detected, suggesting that cross-contamination between pigs of the same and different batches is possible. It is already known that positive pigs can carry *Salmonella* spp. into the abattoir lairage and can contaminate the environment and slaughter pigs from other batches [59]. A period of two hours can be sufficient to infect pigs with *Salmonella* spp. [60]. Three of our 12 *Salmonella* spp. isolates were obtained after the combined process stage of scalding and dehairing. The three pigs involved could have been infected on the farm [61], or the carcasses could have been cross-contaminated by the dehairing process [62].

To classify the different patterns of resistance found in antimicrobial-resistant bacteria, in the literature, there are various different definitions for multiple drug-resistant (MDR), extensively drug-resistant (XDR) and pandrug-resistant (PDR) bacteria [63]. Since the isolate monophasic O-antigen-negative *S.* Typhimurium 1,4,12:i:- showed resistance to ampicillin (antimicrobial class: penicillins), sulfamethoxazole (antimicrobial class: folate pathway inhibitors), and tetracycline (antimicrobial class: tetracyclines) in the antimicrobial susceptibility test, this organism can be characterized as MDR according to a commonly used definition of “resistant to three or more antimicrobial classes” [63,64,65,66,67]. The zoonosis monitoring report 2020 [68] confirmed that resistance to ampicillin, sulfamethoxazole, and tetracycline is frequent in *Salmonella* spp. on pig carcasses in German abattoirs. The finding that only a small percentage (8%, respectively) of the recovered *Salmonella* spp. were resistant to ciprofloxacin and nalidixic acid is in line with the results of the zoonosis monitoring program [68] run by the Federal Office of Consumer Protection and Food Safety (BVL) and the German Federal Institute for Risk Assessment (BfR) in Berlin, Germany.

Our study on the pig slaughter process has the following limitation. The results are specific to this longitudinal study, which was conducted only in a single abattoir, by using the agar contact method on the perianal area. This limits the generalizability and transferability of the results to other abattoirs. However, based on the installed slaughter line machines, it can be presumed that the results are representative for industrial slaughterhouses in Germany with similar slaughter capacities.

## 5. Conclusions

The consideration at the batch level and individual pig level of TVC trend curves determined with the agar contact method showed comparable trends to other previous studies on the pig slaughter process. Moreover, we were able to measure that, factually, the studied process harmonizes the outgoing TVC loads (for batches and individual pigs), regardless of incoming TVC loads, and thus, we were also able to draw conclusions about the functionality/effect of the seven slaughter line process stages. This study shows the importance of studying each process stage in depth and quantifying its effects in the slaughter process, with the aim of understanding and improving each process stage and the ultimate aim of improving pig meat safety. For the implementation of interventions to reduce the TVC on slaughter pig surfaces at the two process stages (after showering in the lairage and after stunning) that produced significant TVC increases, an evaluation should be carried out in further detailed investigations.

Finally, we showed that sampling pig skin via the agar contact method can be used to identify process stages with possible risk factors over the course of the slaughter process and to identify contaminated delivery batches for abattoir-specific testing. However, the agar contact method has the limitation that this particularly animal equitable sampling technique is more suitable for higher expected TVCs, which is the case in the live pig sector at lairage. Nonetheless, in particular for scientific assessment and the optimization of slaughter hygiene, TVCs and *Salmonella* spp. sampled using practical sampling methods, like the agar contact method, are useful, and indeed necessary, for special-case slaughter line examinations.

## Figures and Tables

**Table 1:** Results of the total viable count (TVC log cfu/cm^2^) at the seven process stages.

Process Stage	n	Mean	Median	STD	CV	Min	Max	<LOD
1 Lairage before showering	86	5.70	5.71	±1.05	18.36	3.81	7.95	0
2 Lairage after showering	86	6.27	6.11	±0.72	11.54	4.86	8.00	0
3 After stunning	87	6.48	6.38	±0.70	10.85	4.97	8.15	0
4 After scalding/dehairing	86	3.71	3.49	±0.78	21.10	2.23	6.15	0
5 After singeing/flaming	86	2.70	2.46	±0.86	31.99	1.64	5.57	7
6 After evisceration	87	2.44	2.40	±0.64	26.06	1.64	4.59	14
7 Before chilling	85	2.33	2.20	±0.72	30.69	1.64	4.86	23

n = sample size; STD = standard deviation; CV = coefficient of variation; <LOD = below minimum limit of detection.

**Table 2:** Mean differences of total viable count (TVC) values from one process stage to the following, and *p*-values of the mixed model with the process stage as a fixed effect, batch as a random effect and individual pigs as repeated measurements.

Compared Process Stages	Mean Difference * [TVC log cfu/cm^2^]	*p*-Value
1 Lairage before showering–2 Lairage after showering	0.57	<0.0001
2 Lairage after showering–3 After stunning	0.21	0.0466
3 After stunning–4 After scalding/dehairing	−2.78	<0.0001
4 After scalding/dehairing–5 After singeing/flaming	−1.00	<0.0001
5 After singeing/flaming–6 After evisceration	−0.26	0.0139
6 After evisceration–7 Before chilling	−0.11	0.2891

* = Negative values stand for a reduction from one stage to the following one.

**Table 3:** Detection of *Salmonella* spp. along the slaughter line at seven process stages by depicting isolates of each pig batch and individual pig.

	Process Stage	Stage 1	Stage 2	Stage 3	Stage 4	Stage 5	Stage 6	Stage 7
Pig Batch								
**1**		0	ONST (2 *)	ONST (6 *)	0	0	0	0
**2**		ONST (12 *); ONST (14 *)	ONST (11 *); ONST (18 *)	ONST (17 *)	0	0	0	0
**3**		0	0	0	0	0	0	0
**4**		0	0	0	0	0	0	0
**5**		0	0	0	0	0	0	0
**6**		0	0	MST (51 *)	MST (49 *); MST (53 *); MST (54 *)	0	0	0
**7**		0	0	SP (56 *)	0	0	0	0
**8**		0	0	0	0	0	0	0
**9**		0	0	0	0	0	0	0
**10**		0	0	0	0	0	0	0

Stage 1 = lairage before showering; Stage 2 = lairage after showering; Stage 3 = after stunning; Stage 4 = after scalding/dehairing; Stage 5 = after singeing; Stage 6 = after evisceration; Stage 7 = before chilling; 0 = no detection; * = number of individual pig; ONST = monophasic O-antigen-negative *S.* Typhimurium; MST = monophasic *S.* Typhimurium 1,4,[5],12:i:-; SP = monophasic *Salmonella* spp. 9,12:l,v:-.

**Table 4:** Antimicrobial susceptibility test results for the 12 *Salmonella* spp. isolates, with breakpoints following Clinical and Laboratory Standards Institute (CLSI).

Antimicrobial Agent(s)	Susceptible *		Intermediate *		Resistant *	
	n	%	n	%	n	%
Amikacin	12	100	0	0	0	0
Ampicillin	1	8	0	0	11	92
Azithromycin	12	100	0	0	0	0
Cefotaxime	12	100	0	0	0	0
Ceftazidime	12	100	0	0	0	0
Chloramphenicol	12	100	0	0	0	0
Ciprofloxacin	11	92	0	0	1	8
Colistin	12	100	0	0	0	0
Gentamicin	12	100	0	0	0	0
Meropenem	12	100	0	0	0	0
Nalidixic acid	11	92	0	0	1	8
Sulfamethoxazole	0	0	0	0	12	100
Tetracycline	5	42	0	0	7	58
Tigecycline	12	100	0	0	0	0
Trimethoprim	12	100	0	0	0	0

* = breakpoints susceptibility testing according to the performance standards for antimicrobial susceptibility testing by Clinical and Laboratory Standards Institute (CLSI); n = number of *Salmonella* spp. isolates.

## Data Availability

The data presented in this study are available upon request from the corresponding author.
